# Network proteomic analysis identifies inter-alpha-trypsin inhibitor heavy chain 4 during early human Achilles tendon healing as a prognostic biomarker of good long-term outcomes

**DOI:** 10.3389/fimmu.2023.1191536

**Published:** 2023-07-06

**Authors:** Xinjie Wu, Junyu Chen, Wei Sun, David A. Hart, Paul W. Ackermann, Aisha S. Ahmed

**Affiliations:** ^1^ Division of Spine Surgery, Department of Orthopaedic Surgery, Nanjing Drum Tower Hospital, The Affiliated Hospital of Nanjing University Medical School, Nanjing, China; ^2^ Department of Molecular Medicine and Surgery, Center for Molecular Medicine, Karolinska Institutet, Stockholm, Sweden; ^3^ Department of Orthopedic Surgery, China-Japan Friendship Hospital, Beijing, China; ^4^ Department of Surgery, Faculty of Kinesiology and the McCaig Institute for Bone & Joint Health, University of Calgary, Calgary, AB, Canada; ^5^ Department of Physiology, University of Helsinki, Helsinki, Finland

**Keywords:** connective tissue, Achilles tendon rupture, ITIH4, prognosis, biomarker, therapeutic target

## Abstract

The suboptimal or protracted regeneration of injured connective tissues often results in significant dysfunction, pain, and functional disability. Despite the prevalence of the condition, few studies have been conducted which focused on biomarkers or key molecules involved in processes governing healing outcomes. To gain insight into injured connective tissue repair, and using the Achilles tendon as a model system, we utilized quantitative proteomic and weighted co-expression network analysis of tissues acquired from Achilles tendon rupture (ATR) patients with different outcomes at 1-year postoperatively. Two modules were detected to be associated with prognosis. The initial analysis identified inter-alpha-trypsin inhibitor heavy chain 4 **(**ITIH4) as a biomarker or hub protein positively associated with better healing outcomes. Additional analysis identified the beneficial role of ITIH4 in inflammation, cell viability, apoptosis, proliferation, wound healing, and for the synthesis of type I collagen in cultured fibroblasts. Functionally, the effects of ITIH4 were found to be mediated by peroxisome proliferator-activated receptor gamma (PPARγ) signaling pathways. Taken together, these findings suggest that ITIH4 plays an important role in processes of connective tissue repair and advocate for the potential of ITIH4 as a therapeutic target for injured connective tissue repair.

**Trial registration:**

http://clinicaltrials.gov, identifiers NCT02318472, NCT01317160.

## Introduction

Connective tissues such as tendon and ligament play a wide variety of functions in the joint (1). Moreover, mature connective tissues display impaired capability of regeneration, predisposing joints to degenerative diseases (2). The limited regenerative capability is mainly attributed to a paucity of cells and the relatively avascular or aneuronal nature of the adult tissues, leading to variable and often poor prognosis (3). While the above general perspective of connective tissue healing applies, some patients appear to heal with better outcomes than others (3). Despite such recent advancements, details regarding the pathways and biomarkers governing optimal healing after connective tissue injuries, are mostly unknown and remain to be elucidated.

The healing of connective tissue generally involves the contribution of a variety of cells infiltrating into the site of injury such as macrophages, fibroblasts, as well mesenchymal stem cells (4). Among these, fibroblasts play a crucial role from the early inflammatory to late regenerative healing phase by regulating inflammatory responses, extracellular matrix (ECM) deposition and remodeling as well as specific collagen (Coll) synthesis (5, 6). Collagens are the main component of the ECM and higher collagen type I (Col1) levels at the site of injury are reported as an indicator of better healing after connective tissue injuries (7).

Although connective tissue injuries can occur anywhere in the body, acute Achilles tendon rupture (ATR) is a frequent injury. ATR injuries are becoming more common and with a considerable long associated sick-leave and low frequency of players returning to their previous level of sports activity (8).

In recent years, significant progress has been made in exploring the underlying mechanisms of injured connective tissue healing (9). By using mass spectrometry (MS) based advanced proteomic techniques, our research group has recently identified elongation factor-2 (eEF2) at the early inflammatory healing phase and complement factor D (CFD) at the proliferative healing phase, as potential healing biomarkers predictive of patient outcomes with ATR (3). These biomarkers exhibited differential expression patterns among good and poor outcome patient subgroups. These studies are continuous, using diverse bioinformatic approaches for analysis of the proteomic data.

In the present study, we used a weighted co-expression network analytical approach with the MS proteomic data collected from tissue samples taken at the time of surgery to further characterize the basis for the differences in outcomes between patient subsets. The network-based co-expression analysis of proteomic data approach identifies modules specifically related to the prognosis and subsequently detected prognostic biomarkers or hub proteins. Identified biomarkers/hub proteins were further subjected to Gene ontology (GO) enrichment and Kyoto Encyclopedia of Genes and Genomes (KEGG) pathway analysis to ascertain their biological functions and specific signaling pathways leading to tissue repair. Thus, employing the network proteomic approach may provide new insights regarding specific molecules that may be contributing to more optimal outcomes after endogenous healing of the Achilles tendon.

## Materials and methods

This study was conducted after approval from the Regional Ethical Review Committee in Sweden (Reference no. 2009/2079-31/2: 2013/1791-31/3) and followed all guidelines according to the Declaration of Helsinki. The written informed consent was acquired from all patients.

### Subjects and sampling

Following the inclusion and exclusion criteria as described previously (10), 40 patients with acute ATR who underwent reconstruction surgery with the same surgical protocol were consecutively included in the present study. During the surgery, tendon biopsies were taken from the ruptured area and stored at minus 80^o^ C until proteomic analysis was performed. All the samples were collected within 2-7 days of the ATR injury. Postoperatively, all patients received the same rehabilitation program.

### Patient reported outcomes

Patient-reported outcomes were evaluated 1-year postoperatively using validated questionnaires: Achilles Tendon Total Rupture Score (ATRS). The ATRS consists of 10 sub-scales such as strength in tendon, tiredness in the tendon, stiffness in tendon, pain in tendon, limitations in activity of daily life (ADL) assessing limitations on uneven surface, stairs, running, jumping and loss in physical work (11). Each sub scale ranges from 0 to 10 where 0 = worst and 10 = best outcome with no limitation. The maximum ATRS is 100, and a score higher than 80 was regarded as indication of a good outcome (10).

### Functional outcomes

The functional outcomes were measured using the Heel-rise Test (HRT) at 1-year post-surgery. HRT is a validated test, indicating the outcome of strength and endurance of the affected gastrocnemius-soleus complex (12, 13). The HRT was performed on one leg with the patient standing on a box with a 10° incline. Patients were instructed to perform as many maximal height heel-rises as possible and as many heel-rise repetitions as possible. All the results, including the number of heel-rises, the height of every single heel-rise, the total work in joules (total distance × body weight), the time and the power (work/time) were recorded for analysis. The Limb Symmetry Index (LSI) was used to show the ratio between the injured and contralateral uninjured leg and results are presented in a percentage (injured/contralateral × 100).

### Mass spectrometry

#### Protein extraction and digestion of tissue samples

The methods used were the same as reported previously (3). Frozen samples were powdered by Mikro-dismembrator (B. Braun Biotech International, Germany) on dry ice. Powdered tissue samples were solubilized in 8M urea and 100 mM NaCl with 1% ProteaseMAX (Promega) in 100 mM ammonium bicarbonate (AmBic) and mixed vigorously. Low binding silica beads (400 µm, Ops Diagnostics, Lebanon NJ) were added to each sample and vortexed at high speed. Subsequently, samples were subjected twice to disruption on a Vortex Genie disruptor for 2 min before addition of AmBic, urea and NaCl. Following centrifugation, the 50 mM AmBic was added and vortexed vigorously. Proteins were then reduced with 100 mM dithiothreitol in 50 mM AmBic, incubated at 37°C and alkylated with 100 mM iodoacetamide in 50 mM AmBic. The reaction was stopped with formic acid and the samples were then cleaned on a C18 Hypersep plate (bed volume of 40 µL, Thermo Scientific) and dried in a vacuum concentrator (miVac, Thermo Scientific).

#### Reversed phase liquid chromatographic-MS/MS analysis

Briefly, RPLC of peptides were performed on a C18 EASY-spray and C18 trap columns connected to an Ultimate 3000 UPLC system (ThermoFisher). Mass spectra were acquired on an Q Exactive HF mass spectrometer (ThermoFisher), targeting 5 x 10^6^ ions with maximum injection time of 100 ms, followed by data-dependent higher-energy collisional dissociation (HCD) fragmentations from precursor ions with a charge state.

#### Proteomic data analysis, protein identification and quantification

Raw files were imported to Proteome Discoverer v2.3 (ThermoFisher) and analyzed using the SwissProt protein database with the Mascot v 2.5.1 (MatrixScience Ltd., UK) search engine. MS/MS spectra were matched with The Human Uniport database (last modified: 3 September 2020; ID: UP000005640; 75,777 proteins) using the MSFragger database engine. Protein abundance was calculated based on normalized spectrum intensity (LFQ intensity), and an intensity-based absolute quantification (iBAQ) algorithm was used for normalization.

### Weighed co−expression network analysis

Following previously described procedures of WGCNA (14), a weighted protein co-expression network was generated using the protein abundance network of unique proteins. The soft power applied for gene modules identification was selected to 7. Correlation coefficients between the modules and traits were calculated using Pearson’s method. Prognosis-related protein modules were defined as those with a P value less than 0.05. All proteins from the selected modules were then visualized into a protein-protein interaction (PPI) network using the Cytoscape software (http://cytoscape.org/).

### Functional enrichment analysis

Enrichment analysis of proteins was performed by the ‘clusterProfiler’ package (15). Gene ontology (GO) term analysis consists of biological processes (BP), cellular components (CC), molecular function (MF). Pathway enrichment analysis was conducted based on Kyoto Encyclopedia of Genes and Genomes (KEGG). P < 0.05 was considered statistically significant.

### Identification of hub proteins

The hub proteins of a selected module were determined through an absolute value of the Module Membership (MM) > 0.8 and a Gene Significance (GS) > 0.1. Moreover, the hub proteins in selected modules were calculated and identified using the CytoHubba plugin in the Cytoscape software (16). The common hub proteins identified by the use of three different methods, WGCNA, MCC, and Degree, were investigated further.

### Gene set enrichment analysis

Gene set enrichment analysis (GSEA) was performed by the ‘clusterProfiler’ package (15) to identify enriched hub protein-related signaling pathways and ‘c2.cp.kegg.v7.0.symbols.gmt’ was selected as the reference gene set. A false discovery rate (FDR) < 0.25 and p < 0.05 was considered as significant enrichment.

### Antibodies and reagents

The antibodies used in the present study were as follows: anti-COL1A1 (72026) and anti-PPARγ (C26H12) from Cell Signaling Technologies (Boston, USA); and anti-ITIH4 (ab180139) from Abcam (Cambridge, UK). Secondary antibodies were either goat Alexa 488 (A-11008) or 594 (A-11012) obtained from ThermoFisher (Oxford, UK). Lipopolysaccharide (LPS, L2630) was purchased from Sigma (Steinheim, Germany).

### Cell culture and treatment

The cells of the human dermal fibroblast cell line (fHDF/TERT166) were purchased from Evercyte (Vienna, Austria) and were cultured in Dulbecco’s Modified Eagle Medium/Nutrient Mixture F-12 (DMEM/F-12, 10565018, Gibco) supplemented with 10% fetal bovine serum (FBS) and 1% antibiotics (penicillin-streptomycin) at 37 °CC in a 5% CO_2_ humidified incubator. Different concentrations of LPS diluted in sterilized deionized water, varied from 0.01 μg/ml to 50 μg/ml, were used for cell activation experiments. Experiments were performed in triplicates and repeated at least three times independently.

### Enzyme-linked immunosorbent assay

Enzyme-linked immunosorbent assays (ELISA) were used to investigate the synthesis of ITIH4 and interleukin-6 (IL-6). According to the instructions of the manufacturers, cell culture supernatant levels for ITIH4 (DY8157-05, R&D) and IL-6 (BMS213-2, ThermoFisher) were assessed, respectively. The absorbance was measured at 450 nm using a microplate reader (SpectraMax iD3, Molecular Devices).

### Cell viability assay

The PrestoBlue™ Cell Viability Reagent (A13261, ThermoFisher) was utilized to estimate cell viability at 24hr, 48hr, and 72 hr of culture. PrestoBlue™ solution (10 μL) was added to the cells in each well and incubated at 37 °C for 1 hr. The fluorescence of each well was subsequently evaluated at 560/590 nm using a microplate reader (SpectraMax iD3, Molecular Devices).

### Cell proliferation assay

To confirm the changes in the proliferation rates, the EdU-488 Cell Proliferation Kit (C10337, ThermoFisher) was used according to the manufacturer’s instructions. The results were assessed by fluorescence microscopy (ZOE Cell Imager, Bio-Rad). ImageJ software (NIH, Bethesda, MD, USA) was then used to analyze the cell proliferation rate.

### Apoptosis and caspase activity assay

To determine whether cells were live (green) or dead (red), cells were stained with the LIVE/DEAD Cell Imaging kit (R37601, ThermoFisher) according to the manufacturer’s instructions. Caspase activation was investigated using CellEvent^TM^ Caspase3/7 detection reagent (C10423, ThermoFisher) per the manufacturer’s instructions. Both assays were imaged using fluorescence microscopy (ZOE Cell Imager, Bio-Rad) and analyzed by ImageJ software (NIH, Bethesda, MD, USA).

### In vitro model of wound healing

To observe the migration of the fibroblasts, the *in vitro* wound healing scratch assay was utilized (17). A scratch was created on a cell monolayer by using a 10 μL pipette tip. At 0 h and 24 h, the width of the scratch was assessed, and the rate of scratch recovery was determined. Migration activity was imaged under an optical microscope (ZOE Cell Imager, Bio-Rad) and analyzed by ImageJ software (NIH, Bethesda, MD, USA).

### Immunofluorescence analysis

Briefly, for the immunofluorescence analysis, cells were fixed in 4% paraformaldehyde for 15 min. After permeabilization with 0.1% Triton X-100 for 15 min, the cells were then blocked with 3% bovine serum albumin for 1 hr and were subsequently, incubated with a primary antibody to COL1A1 or PPARγ overnight at 4 °C followed by incubation with fluorescent-conjugated secondary antibodies for 1 hr. The cell nuclei were stained by Hoechst 33342 (ThermoFisher) for 30 min. Images were captured under a fluorescence microscopy (ZOE Cell Imager, Bio-Rad) and analyzed by ImageJ software (NIH, Bethesda, MD, USA).

### Cell transfection

The ITIH4 siRNA and negative controls were obtained from ThermoFisher (AM16708). Lipofectamine™ RNAiMAX Transfection Reagent (13778030, ThermoFisher) was used to introduce siRNA into fibroblasts according to the manufacturer’s protocol. After transfection with ITIH4 siRNA, or scramble control siRNA, fibroblasts were stimulated with LPS.

### Statistical analysis

All statistical analyses were performed using R software (version 4.0.4; R Core Team 2020, Vienna, Austria) or GraphPad Prism software (version 8.0; GraphPad Software Inc., La Jolla, CA, USA). Continuous variables were expressed as the mean ± SD values and compared with the Student’s t-test or one-way analysis of variance. Categorical variables were expressed as frequencies and proportions and compared with the chi-square and Fisher exact tests. Univariate analysis of hub proteins was performed using Logistic regression models to determine the hub proteins associated with prognosis. Receiver Operating Characteristics (ROC) curve analysis and area under the ROC Curve (AUC) was performed and calculated. Meanwhile, the optimal cutoffs were identified by maximizing Youden’s J index. Data with a P value < 0.05 was considered significant.

## Results

### Clinical parameters

**Figure 1:**
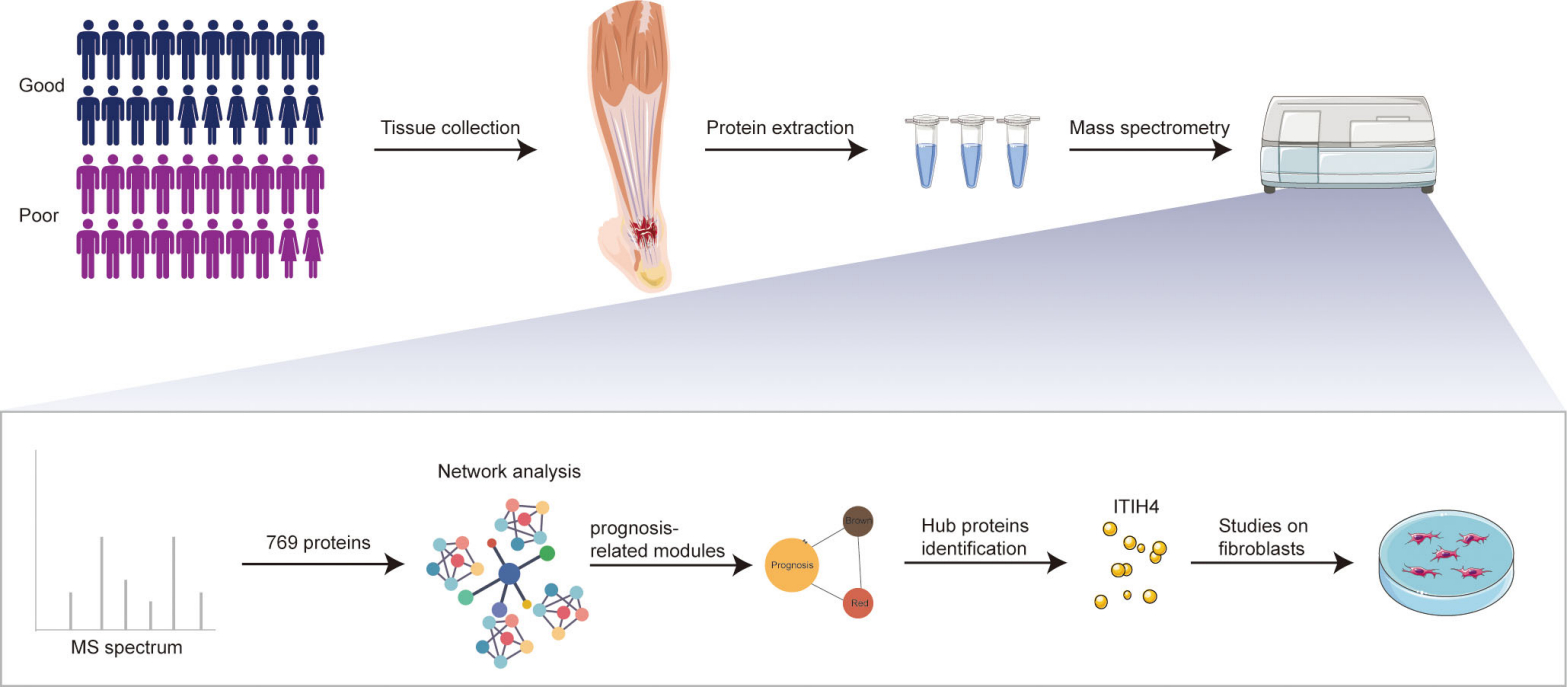
Schematic overview of experimental design. Quantitative proteomic analysis of the tendon tissues from ATR patients with different outcomes was performed based on RPLC-MS/MS, followed by network analysis and related *in vitro* studies on fibroblasts.

The study included a total of 40 patients with ATR undergoing tendon reconstruction surgery. At one-year post-surgery during the follow-up, all patients were assessed for healing outcomes based on their ATRS. To explore the underlying mechanism of connective tissue repair, patients were divided into groups of good (ATRS > 80; n = 20) and poor healing outcomes (ATRS < 80; n = 20). No statistically significant differences were noted between the good and poor outcome groups regarding age, sex, and body mass index. However, the ATRS for the good outcome group (95.3±4.0) was significantly higher than the ATRS for the poor outcome group (61.5±9.67). The clinical parameters for all patients are presented in **Table S1** and the study design is presented in **Figure 1**.

### Proteomics and co−expression protein analysis

**Figure 2:**
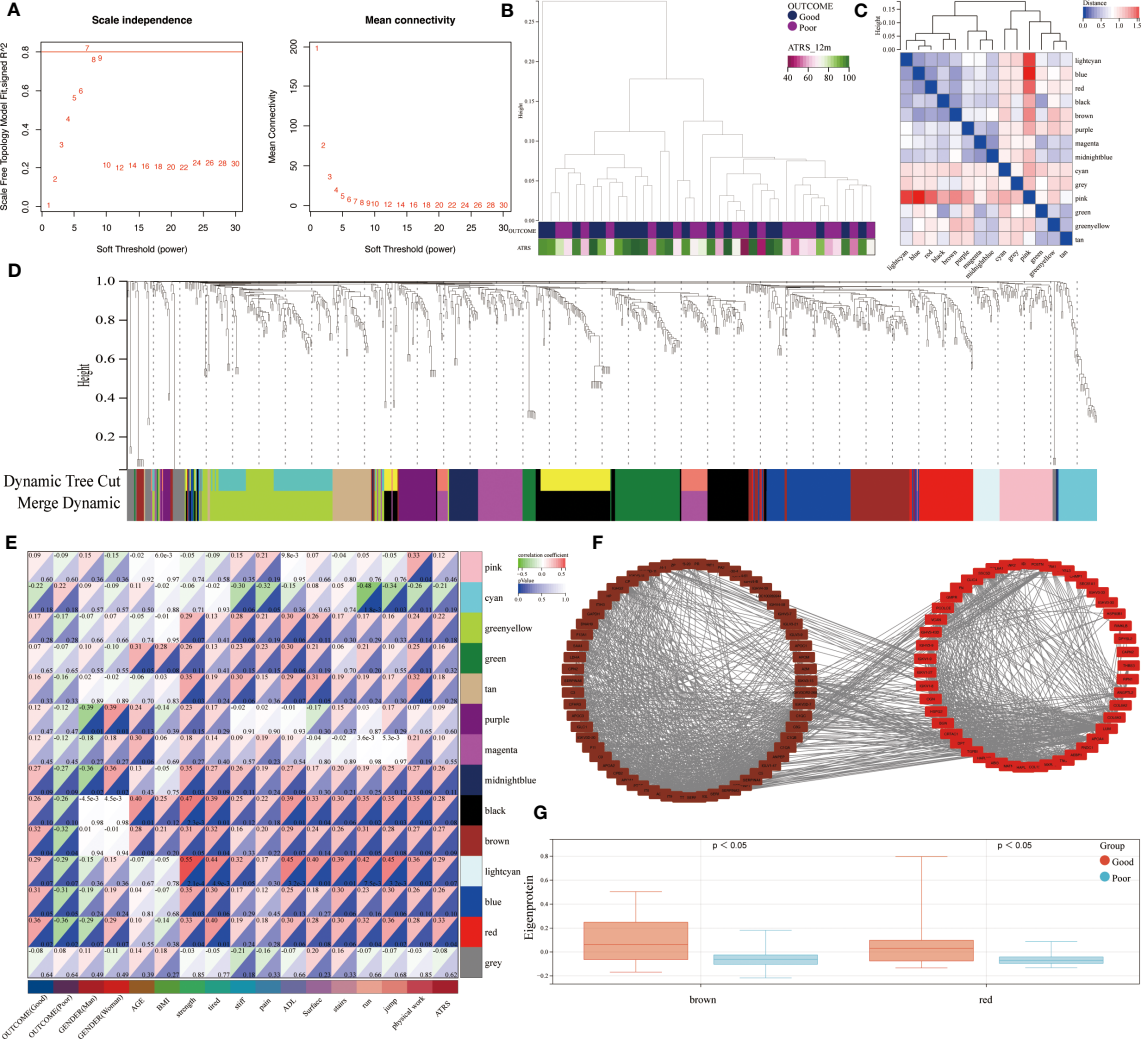
Identification of outcome-related modules and functional enrichment. **(A)** Scale-free topology index and the mean connectivity for each power value between 1 and 30. **(B)** Clustering dendrogram and clinical traits based the expression data between patients with different outcomes. **(C)** Eigengene adjacency heatmap of each module, representing the correlation between the modules. **(D)** Cluster dendrogram of the identified co-expression modules. **(E)** Heatmap of the correlation between modules and clinical traits. Each cell contains the corresponding correlation coefficient and p value. **(F)** Network of brown and red modules. **(G)** The module expressions between patients with different outcomes.

The use of RPLC-MS/MS analysis enabled the identification of a total of 855 unique proteins in all studied patients. These included 769 shared proteins across the good and poor outcome groups. A weighted co-expression network analysis was performed to identify the relationship between the protein abundance and clinical outcome. Using sample clustering to detect outliers, a β value of 7 as the first value that gave an R2 of ≥0.8, was selected as the soft-threshold to fulfill the scale-free approximation criterion (**Figures 2A, B**). A total of 14 strongly co-expressed modules were identified through the dynamic tree cutting method (**Figures 2C, D**). Among these, the brown and red modules were observed to be significantly related to prognosis (**Figures 2E, F**) and were highly expressed in good outcome patients (**Figure 2G**).

### GO enrichment analysis and KEGG pathway analysis

**Figure 3:**
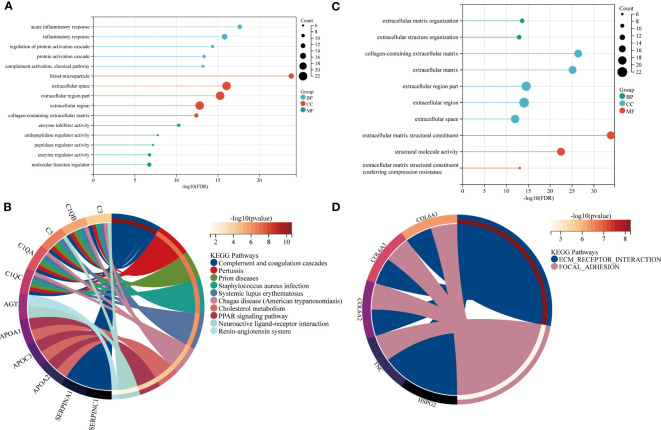
Functional enrichment of brown and red modules. GO and KEGG enrichment analysis of **(A, B)** brown and **(C, D)** red module. Enrichment factor indicating ratio of pathway related proteins in relation to all identified proteomes. Size of bubble represents the number of proteins with the related function. BP = biological process, CC = cellular component, MF = molecular function.

In the next step, the brown and red modules were subjected to further bioinformatic analysis. The functional enrichment analysis revealed that most annotations in the brown module were involved in numerous biological and cellular processes such as inflammation, metabolism, regulation of protein activation, ECM metabolism as well in collagen-containing extracellular matrix and regulation of catalytic activity (**Figure 3A**). Further analysis by KEGG identified enriched signaling pathways which included the complement and coagulation cascades, peroxisome proliferator-activated receptors (PPAR) and cholesterol metabolism (**Figure 3B**).

The enrichment analysis of the red module revealed ECM organization and collagen-containing ECM as the most significant biological and cellular processes. The biological pathways highly enriched in the red module included ECM receptor interaction and focal adhesion. (**Figures 3C, D**).

### Identification of relevant hub proteins and related pathways

**Figure 4:**
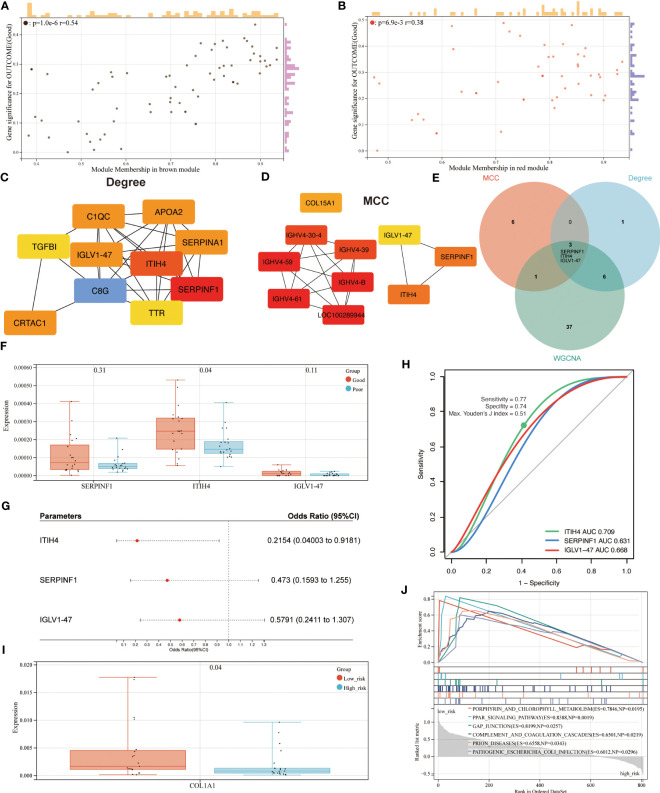
Identification of hub proteins and related pathways. **(A)** Scatterplot of gene significance (GS) versus module membership (MM) for the brown module. **(B)** Scatterplot of GS versus MM for the red module. **(C, D)** Top 10 hub proteins of two modules identified *via* Degree and MCC, respectively. **(E)** Venn diagram of common hub proteins *via* WGCNA, Degree, and MCC methods. **(F)** Synthesis of hub proteins between patients with different outcomes *via* mass spectrum. **(G)** Univariate analysis of hub proteins *via* Logistic regression models **(H)** ROC curve analysis of hub proteins. **(I)** Synthesis of COL1A1 between patients with different risks *via* mass spectrum. **(J)** GSEA analysis of enriched pathways between patients with different risks.

Based on an absolute value of the module membership (MM) > 0.8 and gene significance (GS) > 0.1, 24 hub proteins from the brown and 23 from the red module were selected for further evaluation (**Figures 4A, B**). In the next step, the network was imported into the Cytoscape software and CytoHubba plugin was employed. By using the MCC and Degree methods, the top 10 hub proteins from the brown and red modules were detected along with their interaction as shown in **Figures 4C, D**. Subsequently, three common proteins, inter-alpha-trypsin inhibitor heavy chain 4 (ITIH4), Serpin family F member 1 (SERPINF1), and immunoglobulin lambda variable 1-47 (IGLV1-47), were detected (**Figure 4E**). Among these, the ITIH4 synthesis was found to be elevated in the good- compared to the poor outcome groups (**Figure 4F**). ITIH4, SERPINF1 and IGLV1-47 were further subjected to logistic regression analysis, which also identified a low odds-ratio 0.22 (0.04-0.92) of risk of poor prognosis with ITIH4 (**Figure 4G**). Moreover, the prognostic reliability of ITIH4, SERPINF1 and IGLV1-47 was studied by the ROC curve analysis which demonstrated ITIH4 with the highest area under the curve (AUC) value of 0.71 as a strong predictor of good clinical outcome (**Figure 4H**).

Through maximizing Youden’s J index, the optimal cutoff was identified, and the patients were divided into two groups based on risk levels of poor prognosis (**Figure 4I**). As Col1 is the main collagen subtype of tissue repair, the relationship between ITIH4 and collagen type 1 A1 (COL1A1) was explored further. Our bioinformatic analysis showed elevated COL1A1 levels in the group with low compared to high-risk, indicating the positive effects of ITIH4 levels on collagen synthesis (**Figure 4I**).

To identify the potential pathways for connective tissue repair, GSEA was performed which selected PPAR as the highest ranked signaling pathway with an enrichment score of 0.84 (**Figure 4J**). Taken together, these analyses selected ITIH4 as the best predictive biomarker and PPAR as an associated signaling pathway, findings which were then subjected to further targeted investigations.

### ITIH4 regulates inflammatory responses in human fibroblasts

**Figure 5:**
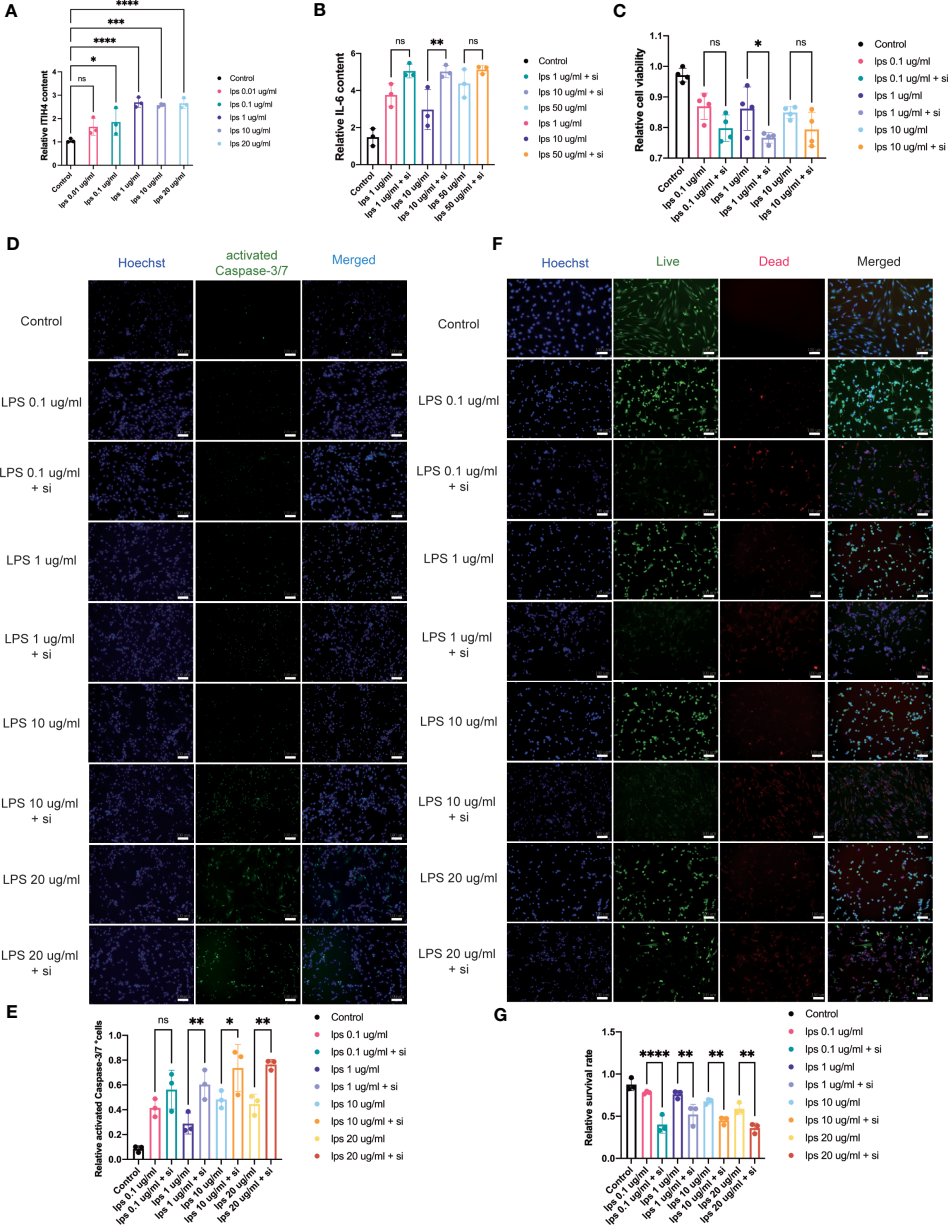
Influence of knockdown of ITIH4 on inflammation, cell viability, and apoptosis of fibroblasts treated with LPS. **(A)** Relative synthesis of ITIH4 investigated through ELISA analysis (n=3). Fibroblasts were transfected with 50 nM ITIH4 siRNA. At 24 h after transfection, fibroblasts were subjected to 24 h of LPS treatment with different concentrations. **(B)** Relative synthesis of IL-6 *via* ELISA analysis (n=3). **(C)** Cell viability assessed by the PrestoBlue™ Cell Viability Reagent (n= 4). **(D, E)** Representative images and quantitative analysis of Caspase-3/7 activation. (n=3). **(F, G)** Representative images and quantitative analysis of cell death assessed by live/dead assay. (n=3). Scale bar = 100 μm. ns, not significant; ^*^ P < 0.05; ^**^ P < 0.01; ^***^ P < 0.001; ^****^ P < 0.0001.

The bioinformatic analysis identified inflammation as one of the crucial biological processes for connective tissue repair especially for annotations from the brown module. To study the role of ITIH4 in intracellular inflammatory processes, ITIH4 synthesis was knocked down by siRNA-ITIH4 in the human fibroblast cell line, fHDF/TERT166 (**Supplementary Figures 1A, B**). The effects of LPS on ITIH4 were then studied and it was observed that LPS treatment elevated ITIH4 levels in a dose-dependent manner (**Figure 5A** and **Supplementary Figures 2 A-C**). Notably, there was a decrease in ITIH4 after simulations of LPS at concentrations of 10 and 20µg/ml after 48 and 72h. This may be attributed to high concentrations of LPS treatment leading to an increase of cell death, which, in turn, decreased the secretion of ITIH4 after 48 and 72 h. In the next step, si-ITIH4 treated fibroblasts were stimulated with LPS and the synthesis of a classical marker of inflammation; IL-6 was assessed. Interestingly, dual effects were observed for both LPS and ITIH4 (**Figure 5B**, **Supplementary Figures 3A, B**) on IL-6 synthesis, highlighting a potential role of this biomarker during the early inflammatory stage of Achilles tendon repair.

### ITIH4 can regulate human fibroblast viability

Metabolic processes are crucial for cell survival as emphasized by the bioinformatic analysis. To determine the potential role of ITIH4 on metabolic activity, cell viability was assessed during LPS-induced inflammatory conditions on the human fibroblasts *in vitro*. The analysis revealed that acute inflammatory responses negatively impact cell viability, while relatively long-term (72 hr) inflammatory responses at lower doses (0.01-0.1 μg/ml) promote cell viability (**Supplementary Figures 4A-C**). Furthermore, the effects of ITIH4 on cell survival were investigated, and the results indicated that knockdown of ITIH4 alone significantly led to decreased cell viability of fibroblasts at 48 and 72 h (10-50 nM) (**Supplementary Figures 4D-F**). Additional analysis revealed that LPS-stimulated fibroblasts with ITIH4 knocked down led to further decreases in cell viability, highlighting a possible protective role for ITIH4 on cell survival and metabolism (**Figure 5C**).

### ITIH4 may regulate connective tissue repair by influencing cell death and apoptosis

To explore a role for ITIH4 in apoptosis, the effect of si-ITIH4 treatment on LPS-induced apoptosis in human fibroblasts was evaluated. The analysis of activated Caspase-3/7 staining demonstrated that LPS alone or knockdown of ITIH4 promoted the activation of Caspase-3/7 in fibroblasts (**Supplementary Figures 5A-D**). During LPS treatments, knockdown of ITIH4 led to higher increases in activation of Caspase-3/7 in the treated fibroblasts than were observed in the LPS group with intact fibroblasts (**Figures 5D, E**). The results were confirmed further by live/dead assays, which showed that treating cells with LPS alone or following knockdown of ITIH4 leads to a decrease in the cell survival rate (**Supplementary Figures 6A-D**). Consistently, following LPS treatments, knockdown of ITIH4 resulted in a higher apoptosis rate (**Figures 5F, G**). Collectively, these results suggest that ITIH4 has a cytoprotective role in LPS-treated fibroblasts.

### ITIH4 may influence inflammatory healing processes via modulation of cell proliferation

**Figure 6:**
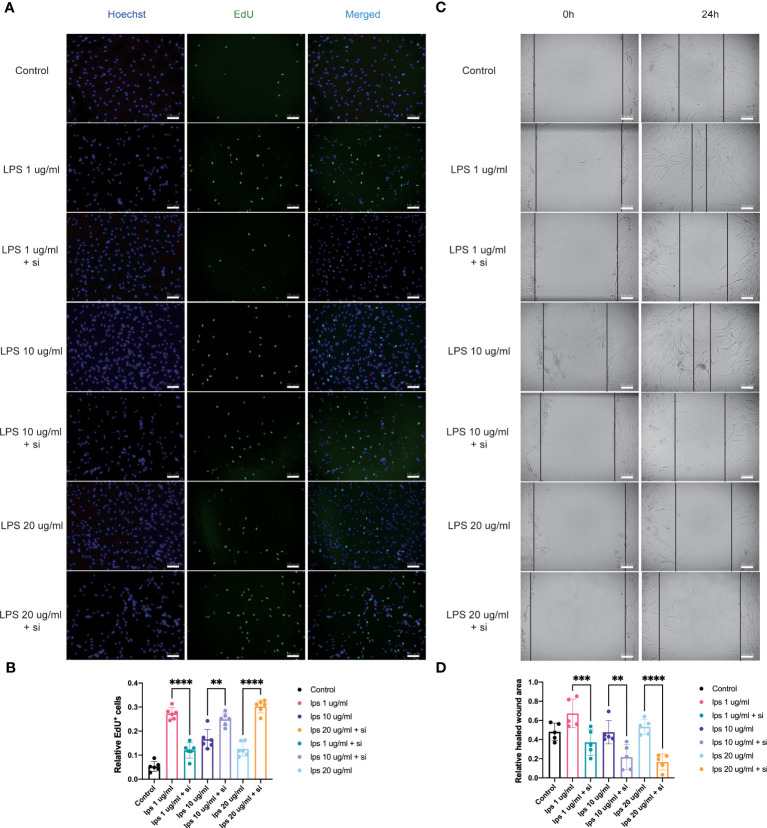
Influence of knockdown of ITIH4 on proliferation and migration of fibroblasts treated with LPS. Fibroblasts were transfected with 50 nM ITIH4 siRNA. At 8 h after transfection, fibroblasts were subjected to 72 h of LPS treatment with different concentrations. **(A, B)** Representative images and quantitative analysis of proliferation rate assessed by EdU assay. (n=6). At 24 h after transfection, a scratch was created, and fibroblasts were subjected to 24 h of LPS treatment with different concentrations. **(C, D)** Representative images and quantitative analysis of cell migration rate assessed *via* a wound healing assay. (n=5). Scale bar = 100 μm. ns, not significant; ^*^ P < 0.05; ^**^ P < 0.01; ^***^ P < 0.001; ^****^ P < 0.0001.

To assess the effect of ITIH4 on human fibroblast proliferative activity, the proliferation rate after ITIH4 knockdown and/or LPS treatment *via* EdU assays was investigated. The results suggested that LPS alone has a pro-proliferative effect in the long term (**Supplementary Figures 7A, B**). Conversely, knockdown of ITIH4 alone leads to reduction in the proliferation rate of the fibroblasts (**Supplementary Figures 7C, D**). Following LPS treatment at a relatively low concentration (0.1μg/ml), knockdown of ITIH4 inhibited the proliferative activity of the cells (**Figures 6A, B**). However, knockdown of ITIH4 enhanced cell proliferative activity when LPS was applied at relatively high concentrations (10-20 μg/ml) (**Figures 6A, B**). In general, the effect of ITIH4 on fibroblasts proliferation appears to dependent on the level of inflammation induced by LPS in this *in vitro* model.

### ITIH4 may affect inflammatory healing processes via influencing cellular migration

Fibroblast migration at the site of injury is an essential process associated with wound healing (18). With the aim to assess the potential involvement of ITIH4 in this process, an inflammatory *in vitro* model of wound healing was used by creating a scratch in the LPS-stimulated monolayer cultures of the fibroblast cell line treated with and without si-ITIH4. It was observed that LPS-stimulation can promote cell migration even at relatively low concentrations (0.1-10 μg/ml), whereas knockdown of ITIH4 inhibits the rate of cell migration (**Supplementary Figures 8A-D**). The experimental observations highlighted a reduced rate of cell migration in LPS-stimulated fibroblasts when treated with si-ITIH4 in comparison to untreated cells (**Figures 6C, D**). These findings further support a potential beneficial role for ITIH4 in wound healing during the inflammatory phase of healing.

### ITIH4 can regulate collagen synthesis by human fibroblasts

**Figure 7:**
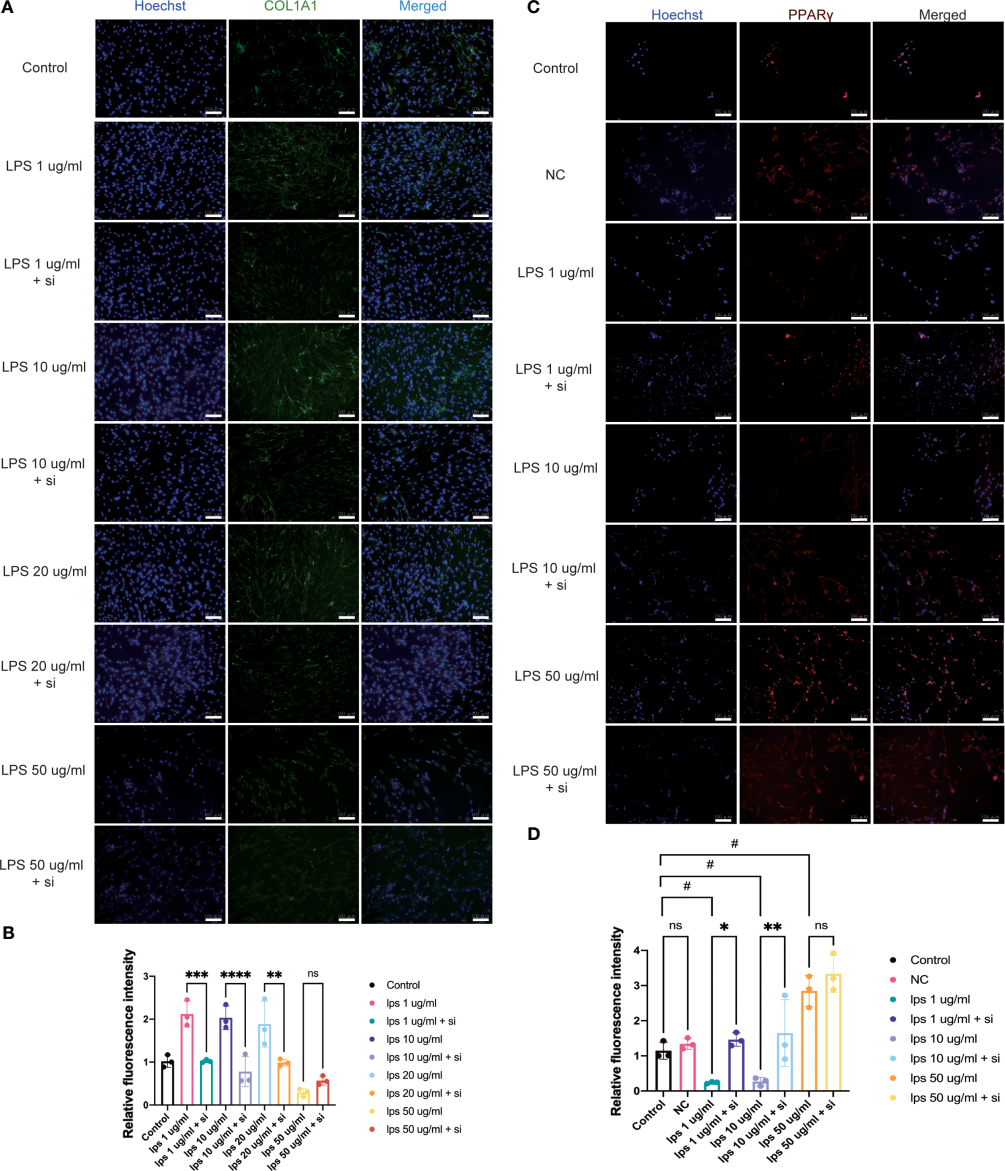
Effect of knockdown of ITIH4 on levels of collagen I and PPARγ in fibroblasts treated with LPS. Fibroblasts were transfected with 50 nM ITIH4 siRNA. At 8 h after transfection, fibroblasts were subjected to 48 h of LPS treatment with different concentrations. **(A, B)** Representative images and quantitative analysis of synthesis of COL1A1 *via* immunofluorescence analysis. (n=3). **(C, D)** Representative images and quantitative analysis of synthesis of PPARγ via immunofluorescence analysis. (n=3). Scale bar = 100 μm. ns, not significant; ^*,#^ P < 0.05; ^**^ P < 0.01; ^***^ P < 0.001; ^****^ P < 0.0001.

Col1 is the main constituent of the connective tissue matrix and an essential component of the ECM organization. The bioinformatic analysis highlighted the process of collagen containing matrix production as one of the most enriched biological processes leading to a good healing outcome (**Figures 3A, C**). Additionally, patients with higher ITIH4 synthesis exhibited elevated COL1A1 levels in surgical biopsies (**Figure 4I**). To further evaluate the effect of ITIH4 on Col1 synthesis during the inflammatory stage of healing, analysis of anti-Col1 staining using immunofluorescence was performed. The results indicated that relatively, low grade inflammation induced by LPS (0.1-20 μg/ml) can lead to an upregulation of COL1A1 synthesis whereas higher LPS concentrations (50 μg/ml) has a negative impact on COL1A1 synthesis (**Supplementary Figures 9A, B**). Interestingly, ITIH4 knockdown by si-ITIH4 alone was observed to reduce COL1A1 synthesis (**Supplementary Figures 9C, D**). Following LPS treatments, knockdown of ITIH4 suppressed the beneficial effects of LPS, leading to a downregulation of COL1A1 synthesis (**Figures 7A, B**). Thus, ITIH4 synthesis is positively correlated with Col1A1 synthesis in these cells.

### Association between ITIH4 and the PPAR signaling pathway

The bioinformatic analysis identified PPAR as the most highly ranked signaling pathway associated with good healing outcomes after ATR. To confirm a potential association between PPAR and ITIH4 synthesis, immunofluorescence analysis with anti-ITIH4 and anti-PPAR-gamma was used with LPS-stimulated fibroblasts in the presence or absence of ITIH4. The results showed that LPS treatment alone downregulated the synthesis of PPARγ at relatively low concentrations (1-10 μg/ml), while at a relatively high concentration (50 μg/ml) it upregulated PPARγ synthesis. However, the knockdown of ITIH4 by si-ITIH4 significantly increased PPARγ synthesis in LPS-stimulated fibroblasts (**Figures 7C, D**). Thus, the presence or absence of ITIH4 can impact synthesis of components of the PPAR pathway.

## Discussion

In the present studies, we have identified prognostic biomarkers and pathways of connective tissue repair by using in-depth proteomic and co−expression network analysis of MS data acquired from ruptured human Achilles tendon tissues early after injury. A total 14 modules were identified from which 2 were determined to be significantly related to healing prognosis after ATR. Further analysis of elements in these prognostic modules identified ITIH4 as a prominent biomarker associated with better patient-reported outcomes. Functionally, the beneficial effect of ITIH4 on Col1 synthesis were observed to be potentially mediated by the PPARγ signaling pathway.

The first major finding was that ITIH4, a 120 KDa glycoprotein that is a component of the inter-alpha trypsin inhibitor, is a potential biomarker and hub protein prognostic of long-term Achilles tendon healing. Previously, prognostic roles for elevated ITIH4 synthesis have been identified in inflammatory and infectious diseases with an elevated ITIH4 synthesis (19). In addition, higher synthesis of ITIH4 in patients with chronic hepatitis B-virus or HBV-related hepatocellular carcinoma patients was reported to be related to good prognostic outcomes in these diseases (20, 21). Here, by using advanced MS techniques along with deep bioinformatics, a prognostic role for ITIH4 in human Achilles tendon healing with good sensitivity and specificity was identified.

Functionally, a role for ITIH4 has previously been reported in liver metabolic processes, as well in inflammatory processes and neutrophil migration in arthritic joints (19, 22–24). As well, ITIH4 has also been reported to be a serologic biomarker in rheumatoid arthritis (25). In these disorders, the role of ITIH4 as an acute phase protein has been highlighted. Recently, the function of ITIH4 as a protease inhibitor has been reported, leading to inhibition of a lectin pathway of complement (26). Interestingly, these biological functions were also found to be highly enriched in the identified red and brown modules of the current studies, two modules identified as prognosis-related from the present bioinformatic analysis.

The process of connective tissue repair is often divided into overlapping phases of inflammation, proliferation, provisional matrix deposition, and remodeling. Notably, in the present study, connective tissues from ATR patients could only be collected from the early inflammatory phase at the time of surgery due to ethical considerations. During this stage, higher synthesis levels for ITIH4 in injured tissues were indicative of better recovery outcomes. Interestingly, previous studies have shown that ITIH4 also has a close association with IL-6 and LPS (19, 22). Consistent with those studies, we have demonstrated that in LPS-stimulated fibroblasts, ITIH4 synthesis was increased in a dose-dependent manner. Meanwhile, the secretion of IL-6, was promoted by ITIH4 deficiency. These findings are also consistent with previous observations that higher IL-6 levels delay the healing processes and negatively impact tissue regeneration (27, 28). It was further demonstrated that knockdown of ITIH4 aggravated LPS-induced inflammation, reducing cell viability and apoptosis. Taken together, these findings support a beneficial role for ITIH4 in healing, in part, by regulating IL-6 synthesis during the acute inflammatory phase of connective tissue repair after injury.

A role for ITIH4 in cell growth and regeneration has also been demonstrated in recent publications (29, 30). Consistent with such roles, our bioinformatic analysis identified various proteins that were enriched with metabolism-related annotations. To confirm the bioinformatic analysis, EdU assays were utilized to investigate the effect of ITIH4 on cell proliferation. Moreover, the experimental findings indicated that LPS alone can promote cell proliferation in accordance with previous publications (31, 32). Interestingly, in fibroblasts treated with different concentrations of LPS, knockdown of ITIH4 showed opposing roles on cell proliferation. The results of our studies may potentially be attributed to the presence of different isoforms of ITIH4 which we could not identify from the current methodologic approach. A previous study demonstrated that two isoforms of ITIH4 possess different functions on cell proliferation (22). The authors revealed that the long isoform of ITIH4 significantly inhibits cell proliferation, whereas the short isoform had the opposite effect (22). Taken together, the effect of ITIH4 on cell proliferation may depend on the level of inflammation as well as the isoform of ITIH4 that is present at the site. However, this conclusion is still in need of further confirmation with regards to Achilles tendon healing.

To mimic the inflammatory phase of healing, an inflammatory *in vitro* wound healing model was used. In accordance with previous publications (33, 34), the results obtained also showed that LPS may promote wound healing in a dose-dependent manner. It has been demonstrated that LPS treatment can potentially affect the healing processes by accelerating the resolution of inflammation, increasing immune infiltration, and altering the secretion of a number of mediators (35, 36). However, the beneficial effects of relatively low concentrations of LPS could be reduced *via* knockdown of ITIH4. Thus, the present results revealed that ITIH4 can potentially have positive effects on early wound healing.

In patients with a connective tissue injury such as an ATR, there is an initial reduction of type I collagen, a molecule responsible in part for the reduced tensile strength of the new deposited scar tissues due to lack of molecular cross links (37) as well as the organization of the collagen (38). In the present studies, ECM and collagen-related annotations were highly enriched. In addition, the synthesis of COL1A1 was higher in patients with low risk compared to high risk regarding outcomes. In cultured fibroblasts treated with LPS, the synthesis of COL1A1 was regulated in a dose dependent manner. These results are consistent with previous studies (31). However, in the present studies, the beneficial effects induced by relatively low LPS-concentrations were weakened by knockdown of ITIH4. Interestingly, we found that ITIH4 knockdown did not only result in low synthesis but also different distributions of COL1A1. However, this should be explained with caution. Col I is both located in cytoplasm and extracellular compartments. This may be attributed to various time periods, which still need further research.

According to previous studies, ITIH4 also plays a crucial role in ECM stabilization (39, 40). Hence, the present findings confirm the potential effect of ITIH4 on ECM following exposure to inflammatory environments. These findings also highlight ITIH4 as an integral part of the network of proteins and pathways essential of good healing outcomes.

From the present proteomic studies, the PPAR signaling pathway is highly involved in prognosis for patients with different outcomes. The PPAR pathway is known to be involved in lipid catabolism, inflammation, survival, proliferation, as well as regeneration of the skin, bone and liver (41–43). It has been demonstrated to be an emerging target to promote wound healing and regeneration. PPARs consist of ligand-activated transcription factors belonging to the nuclear hormone receptor super-family (44). As an important member of the super-family, it has been reported that the synthesis of PPAR-gamma is increased following exposure to inflammation, and it significantly suppresses collagen production (45). Relevant to the present discussion, PPAR pathway regulating drugs have been reported to influence wound healing in preclinical models (46). In addition, PPAR-gamma agonists have also recently been documented to regulate ECM production in human fibroblasts (47). In the present studies, the synthesis of PPAR-gamma was regulated by LPS in a dose-dependent manner. Moreover, the synthesis of ITIH4 was negatively correlated with PPAR-gamma in cultured fibroblasts. In the context of LPS-induced inflammation, ITIH4 may act as a negative regulator of PPAR. While moderate or low inflammation upregulate ITIH4 and collagen by downregulating PPAR signaling pathway. Taken together, these results suggest that ITIH4 could regulate type I collagen production *via* regulating the synthesis of PPAR-gamma in inflammatory environments. However, further studies are needed to clarify the association among ITIH4 and PPAR signaling pathway.

In summary, the findings presented provide several molecular and functional insights into the healing mechanisms after connective tissue injuries, and the long-term quality of specific molecules and pathways. Specifically, they provide substantial evidence that ITIH4 can participate in the regulation of inflammatory and proliferating healing processes most probably through PPAR as a signaling pathway. As such, ITIH4 may represent a prognostic biomarker and therapeutic target for effective connective tissue repair and regeneration. ITIH4 may be one of several biomarkers of good versus poor outcomes of healing as the processes are quite complex and application of different methodological approaches may identify different biomarkers. In the future, integration of the knowledge gained from the identification of multiple biomarkers of good outcomes will not only lead to better understanding of the healing processes, but will also enhance opportunities to intervene with those patients destined for a poor outcome to convert the healing process to yield an improved outcome, particularly since the basis for a poor outcome may be due to multiple variables.

## Study limitations

The present study has several limitations. Firstly, due to the technic problem and relatively low expressions of unique proteins, only the common proteins between the two groups were investigated. Further research would be needed to identify and validate certain proteins solely synthesized in good or poor healers. Secondly, due to a relatively small sample size, only univariate analysis of hub proteins was performed using logistic regression models. Future studies with larger sample sizes cohorts should be performed to confirm the role of specific hub proteins and the biomarker identified in our present studies. Secondly, although the *in vitro* studies supported the bioinformatic findings, more advanced *in vivo* experiments based on animal models, or the primary cells extracted from patients with connective injuries, specifically human primary tenocytes, should be used to assess the potential roles of ITIH4 in the healing process. Thirdly, the opposing roles of ITIH4 on proliferation at different inflammatory levels are quite interesting and needs further exploration to elucidate the molecular mechanisms involved. In addition, several signaling pathways are involved in connective tissue healing, however, the PPAR signaling pathway was selected for further investigation due to its high enrichment score. Nevertheless, other pathways likely should also be further investigated for their contributions, investigation that may also provide new insights into connective tissue regenerative processes leading to better outcomes.

## Data availability statement

The datasets presented in this study can be found in online repositories. The names of the repository/repositories and accession number(s) can be found below: PXD033163 (ProteomeXchange)”.

## Ethics statement

The studies involving human participants were reviewed and approved by Regional Ethical Review Committee in Sweden (Reference no. 2009/2079-31/2: 2013/1791-31/3). The patients/participants provided their written informed consent to participate in this study.

## Author contributions

XW participated in the study design, interpretation of data, statistical analysis, experiments, manuscript writing and reviewing. JC performed part of experiments and participated in data collection. WS participated in reviewing of the manuscript. DAH participated in the interpretation of data and reviewed and revised the manuscript. PWA and ASA participated in the study concept and design, interpretation of data, reviewed, and revised the manuscript. All authors contributed to the article and approved the submitted version.
