# Supplementary material for: Network proteomic analysis identifies inter-alpha-trypsin inhibitor heavy chain 4 during early human Achilles tendon healing as a prognostic biomarker of good long-term outcomes
Source: Front Immunol. 2023 Jul 6;14:1191536. doi: 10.3389/fimmu.2023.1191536 (PMC10358850; doi:10.3389/fimmu.2023.1191536)
Supplement: Supplementary file 1 [file DataSheet_1.pdf]

Table legends

Table S1. Patient characteristics and outcome data

Characteristics	Good outcome (N=20)	Poor outcome (N=20)	Total (N=40)	pvalue
**Gender**				ns
Male	14(35.00%)	18(45.00%)	32(80.00%)	
Female	6(15.00%)	2(5.00%)	8(20.00%)	
**Age**	40.55±8.44	41.85±8.70	41.20±8.49	ns
**BMI**	25.47±3.46	25.54±2.65	25.50±3.05	ns
**Limitation in calf strength**	9.00±0.97	5.13±1.81	7.24±2.40	<0.001
**Tiredness in the calf**	9.39±0.98	5.00±1.85	7.39±2.63	<0.001
**Stiffness in the calf**	9.28±1.23	5.20±1.70	7.42±2.51	<0.001
**Pain in the calf**	9.89±0.32	7.93±1.91	9.00±1.62	<0.001
**Activity of daily life**	9.89±0.32	6.93±1.83	8.55±1.94	<0.01
**Walking on uneven surface**	9.72±0.75	7.07±2.37	8.52±2.14	<0.01
**Limitation on walking in stairs**	9.83±0.38	7.20±2.18	8.64±1.98	<0.001
**Limitation on running**	9.67±0.69	4.60±2.10	7.36±2.96	<0.001
**Limitation on jumping**	9.17±1.04	4.00±2.17	6.82±3.08	<0.001
**loss in physical work**	9.89±0.32	6.80±2.08	8.48±2.09	<0.001
**ATRS**	95.33±3.90	61.50±9.67	78.41±18.61	<0.001

Note: BMI, body mass index; **ATRS,** achilles tendon total rupture score; **ns**, not significant;

Figure legends

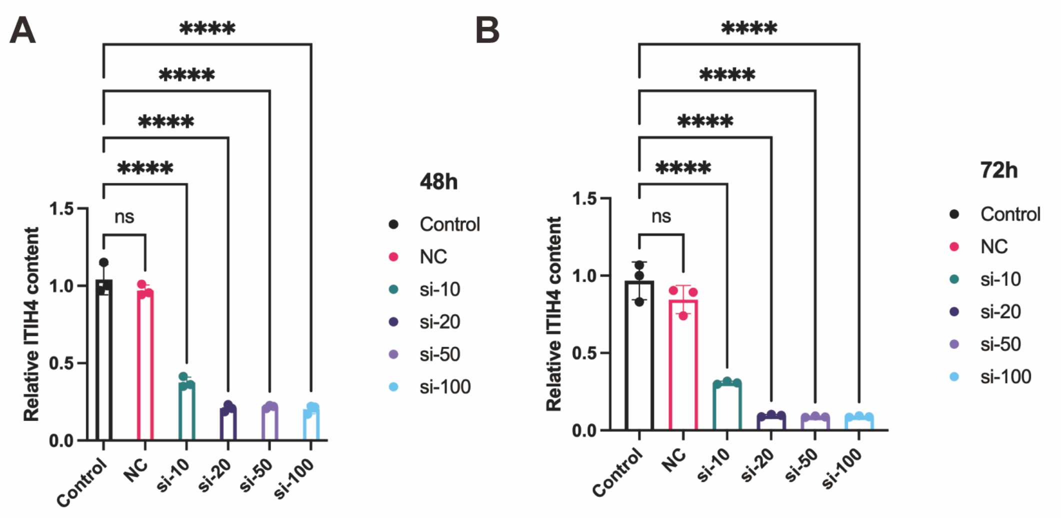

Figure S1. Effect of siRNA on synthesis of ITIH4 in fibroblasts. (A-B) Fibroblasts were transfected with NC siRNA or 10-100 nM ITIH4 siRNA for 48 and 72h, then relative synthesis of ITIH4 was investigated through ELISA analysis (n= 3). ns, not significant; * P ＜ 0.05; ** P ＜ 0.01; *** P ＜ 0.001; **** P ＜ 0.0001

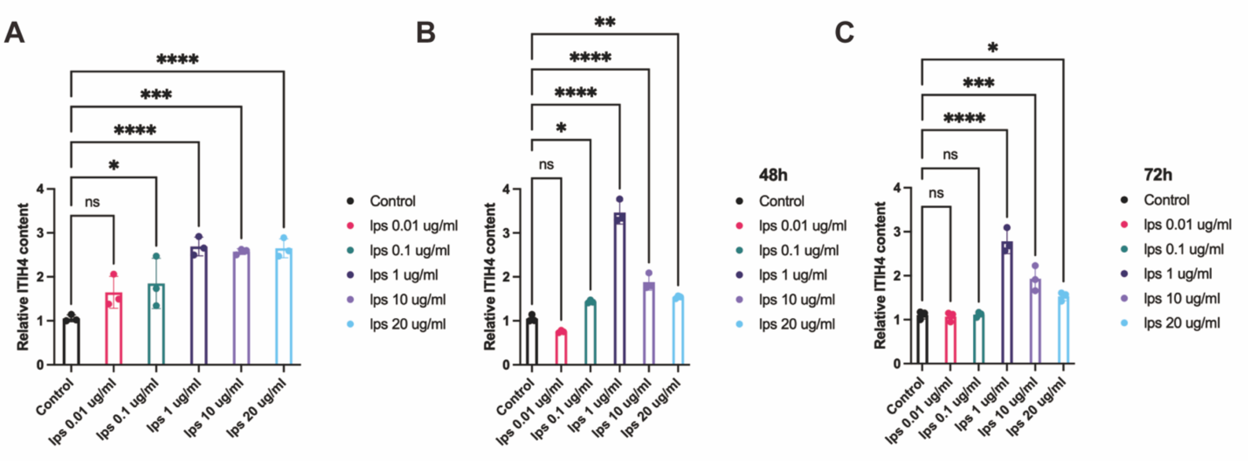

Figure S2. Effect of LPS on synthesis of ITIH4 in fibroblasts. (A-C) Fibroblasts were subjected to 0.1-20 μg/ml LPS treatment for 24 h, 48h, and 72h, then relative synthesis of ITIH4 was investigated through ELISA analysis (n=3). ns, not significant; * P ＜ 0.05; ** P ＜ 0.01; *** P ＜ 0.001; **** P ＜ 0.0001

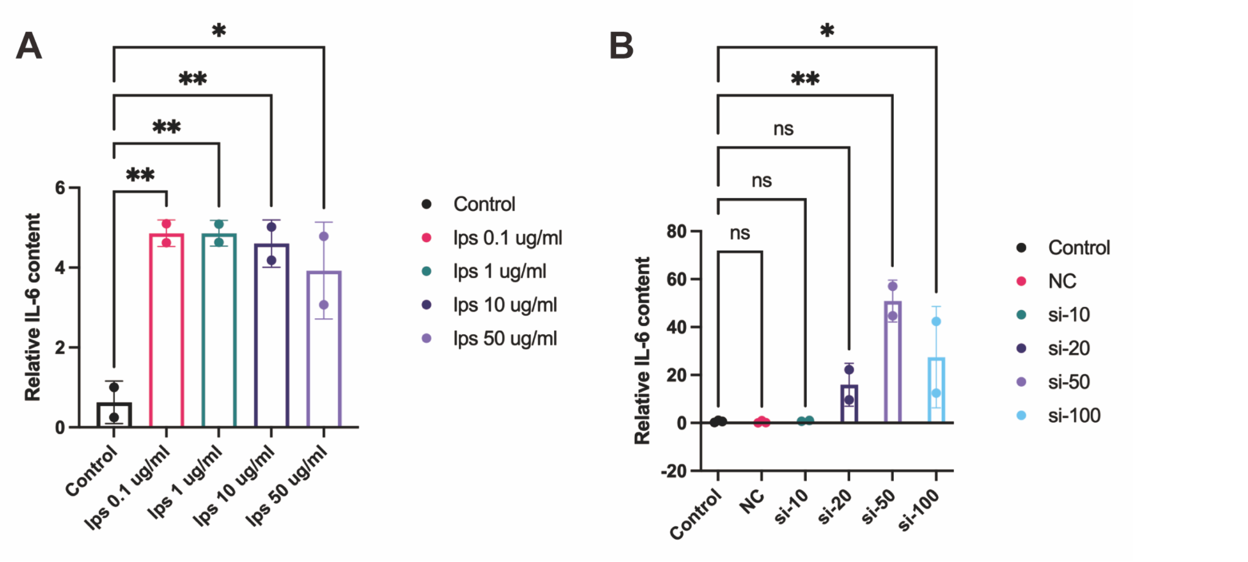

Figure S3. Effect of LPS or knockdown of ITIH4 on inflammation of fibroblasts. (A) Fibroblasts were subjected to 0.1-50 μg/ml LPS treatment for 24 h, then relative synthesis of IL-6 was investigated through ELISA analysis (n=2). **(B)** Fibroblasts were transfected with 10-100 nM ITIH4 siRNA for 48 h, then relative synthesis of IL-6 was investigated through ELISA analysis (n=2). ns, not significant; * P < 0.05; ** P < 0.01; *** P < 0.001; **** P < 0.0001

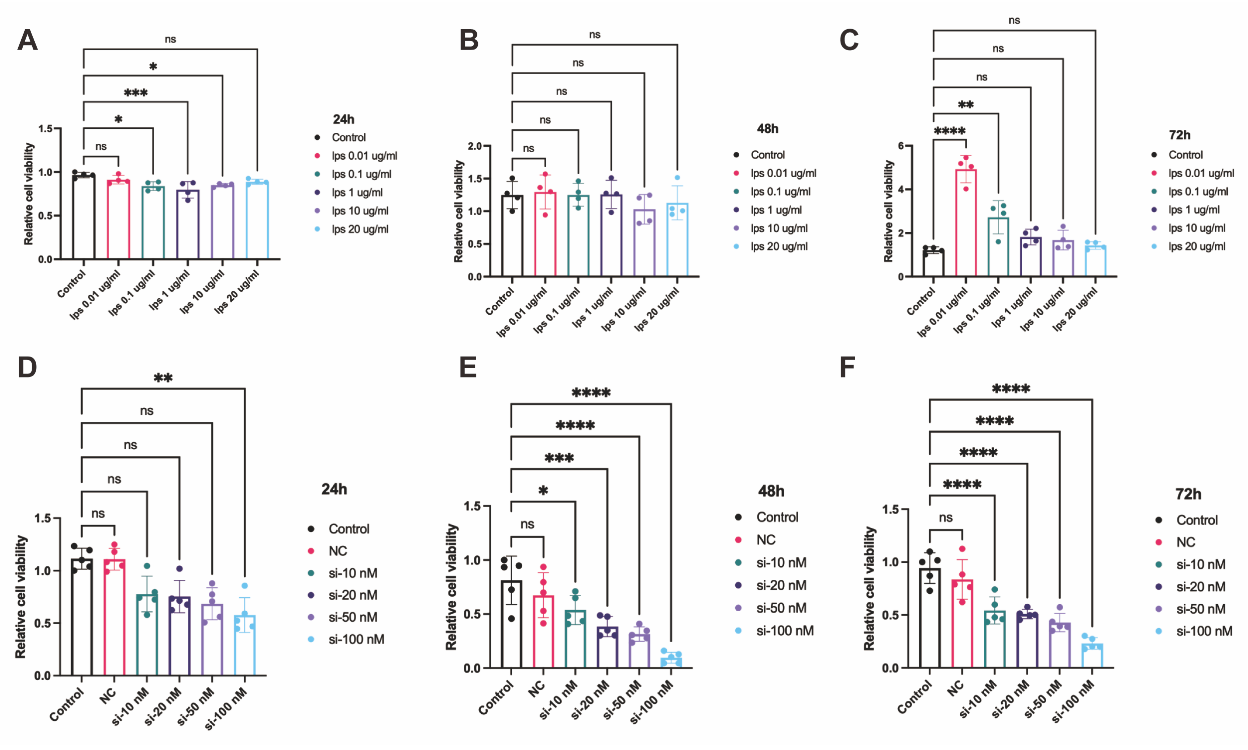

Figure S4. Effect of LPS or knockdown of ITIH4 on cell viability of fibroblasts. (A-C) Fibroblasts were subjected to 0.1-20 μg/ml LPS treatment for 24 h, 48 h and 72 h, then cell viabitliy was assessd by the PrestoBlue™ Cell Viability Reagent (n= 4). **(D-E)** Fibroblasts were transfected with 10-100 nM ITIH4 siRNA for 24 h, 48, and 72h, then cell viabitliy was assessd by the PrestoBlue™ Cell Viability Reagent (n= 5). ns, not significant; * P < 0.05; ** P < 0.01; *** P < 0.001; **** P < 0.0001

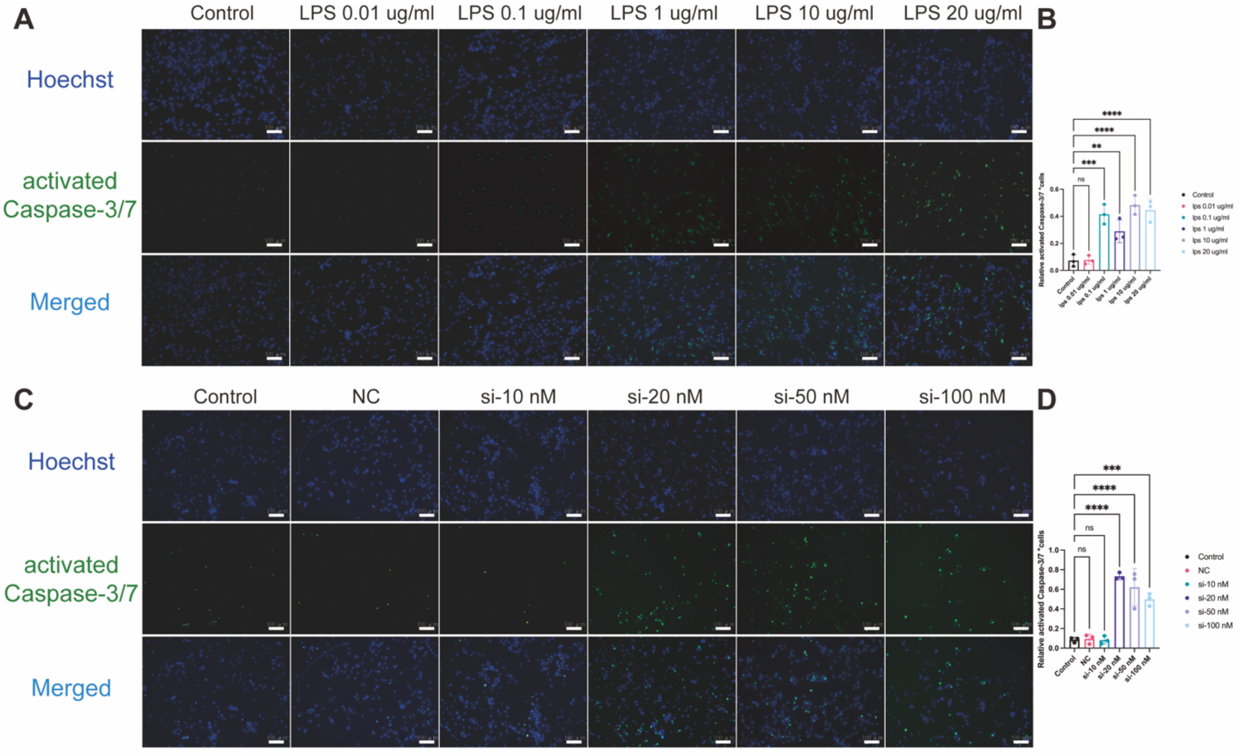

Figure S5. Effect of LPS or knockdown of ITIH4 on Caspase-3/7 activation of fibroblasts. (A-B) Fibroblasts were subjected to 0.01-20 μg/ml LPS treatment for 24 h. Representative images and quantitative analysis of Caspase-3/7 activation. (n=3). **(C-D)** Fibroblasts were transfected with 10-100 nM ITIH4 siRNA for 48 h. Representative images and quantitative analysis of Caspase-3/7 activation. (n=3). Scale bar = 100 μm. ns, not significant; * P < 0.05; ** P < 0.01; *** P < 0.001; **** P < 0.0001

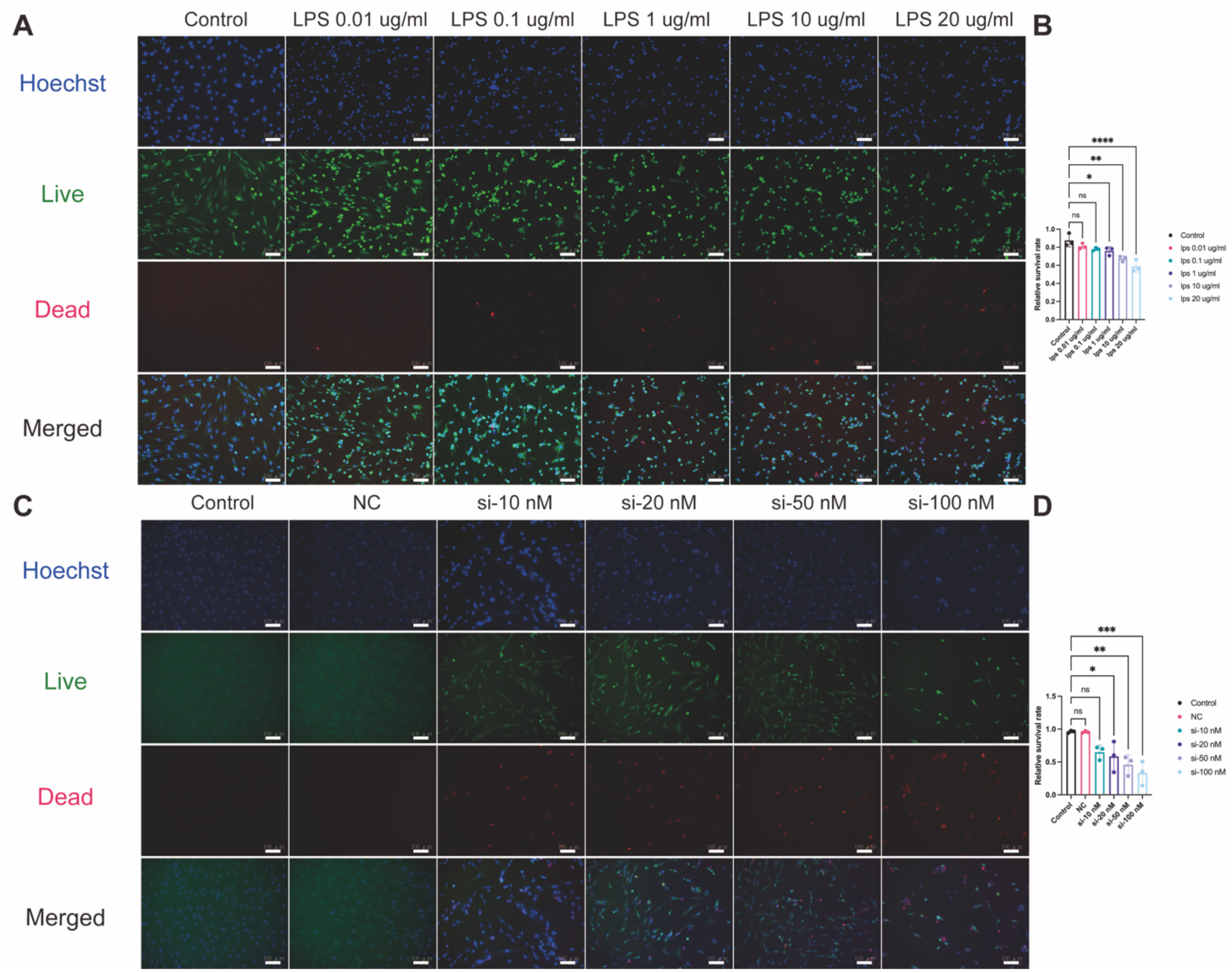

Figure S6. Effect of LPS or knockdown of ITIH4 on cel death of fibroblasts. (A-B) Fibroblasts were subjected to 0.01-20 μg/ml LPS treatment for 24 h. Representative images and quantitative analysis of cell death assessed by live/dead assay. (n=3). **(C-D)** Fibroblasts were transfected with 10-100 nM ITIH4 siRNA for 48 h. Representative images and quantitative analysis of cell death assessed by live/dead assay. (n=3). Scale bar = 100 μm. ns, not significant; $^{*}P < 0.05$; $^{**}P < 0.01$; $^{***}P < 0.001$; $^{****}P < 0.0001$

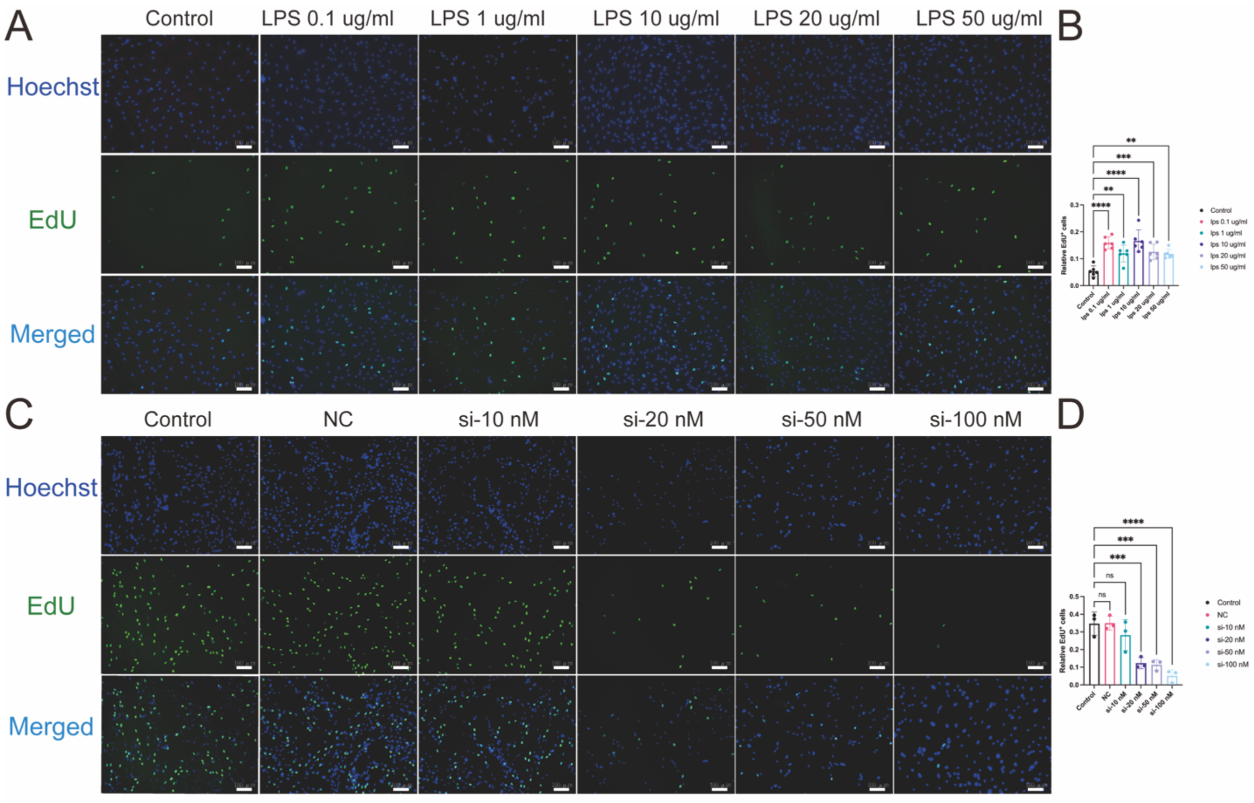

Figure S7. Effect of LPS or knockdown of ITIH4 on proliferation of fibroblasts. (A-B) Fibroblasts were subjected to 0.1-50 μg/ml LPS treatment for 72 h. Representative images and quantitative analysis of proliferation rate assessed by EdU assay. (n=6). **(C-D)** Fibroblasts were transfected with 10-100 nM ITIH4 siRNA for 72 h. Representative images and quantitative analysis of proliferation rate assessed by EdU assay. (n=3). Scale bar = 100 μm. ns, not significant; $^{*}P < 0.05$; $^{**}P < 0.01$; $^{***}P < 0.001$; $^{****}P < 0.0001$

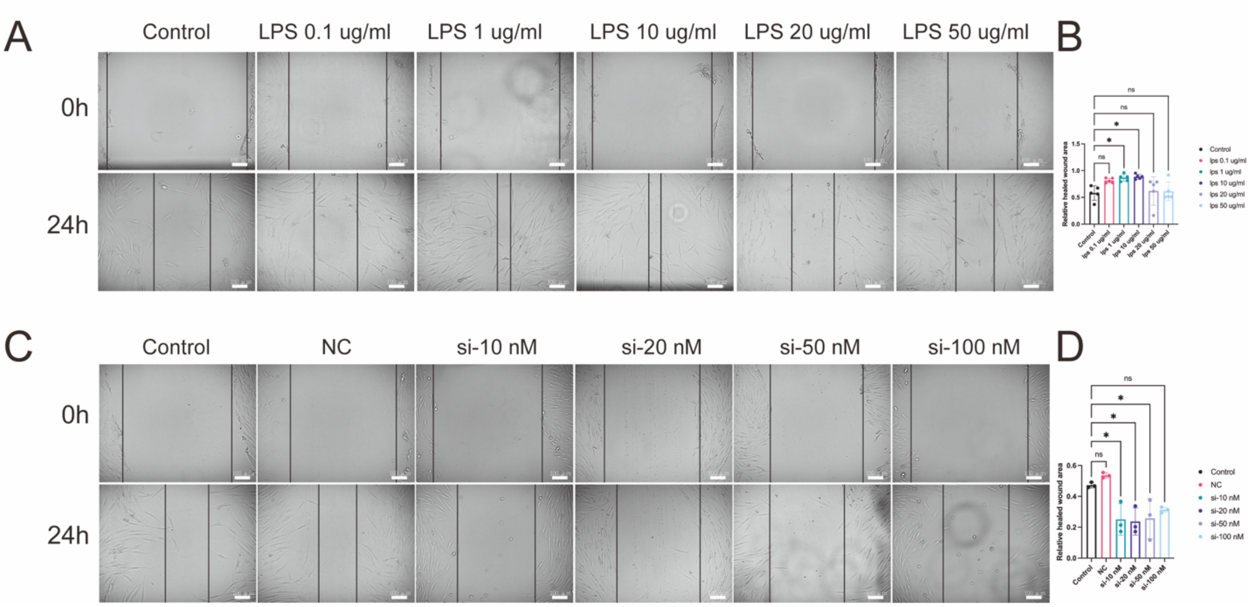

Figure S8. Effect of LPS or knockdown of ITIH4 on wound healing of fibroblasts. (A-B) Fibroblasts were subjected to 0.1-50 μg/ml LPS treatment for 24 h. Representative images and quantitative analysis of wound revcovery rate assessed by wound healing assay. (n=5)**. (C-D)** Fibroblasts were transfected with 10-100 nM ITIH4 siRNA for 24 h, a scratch then was created and observed for 24 h. Representative images and quantitative analysis of proliferation rate assessed by EdU assay. (n=3). Scale bar = 100 μm. ns, not significant; * $P < 0.05$; ** $P < 0.01$; *** $P < 0.001$; **** $P < 0.0001$

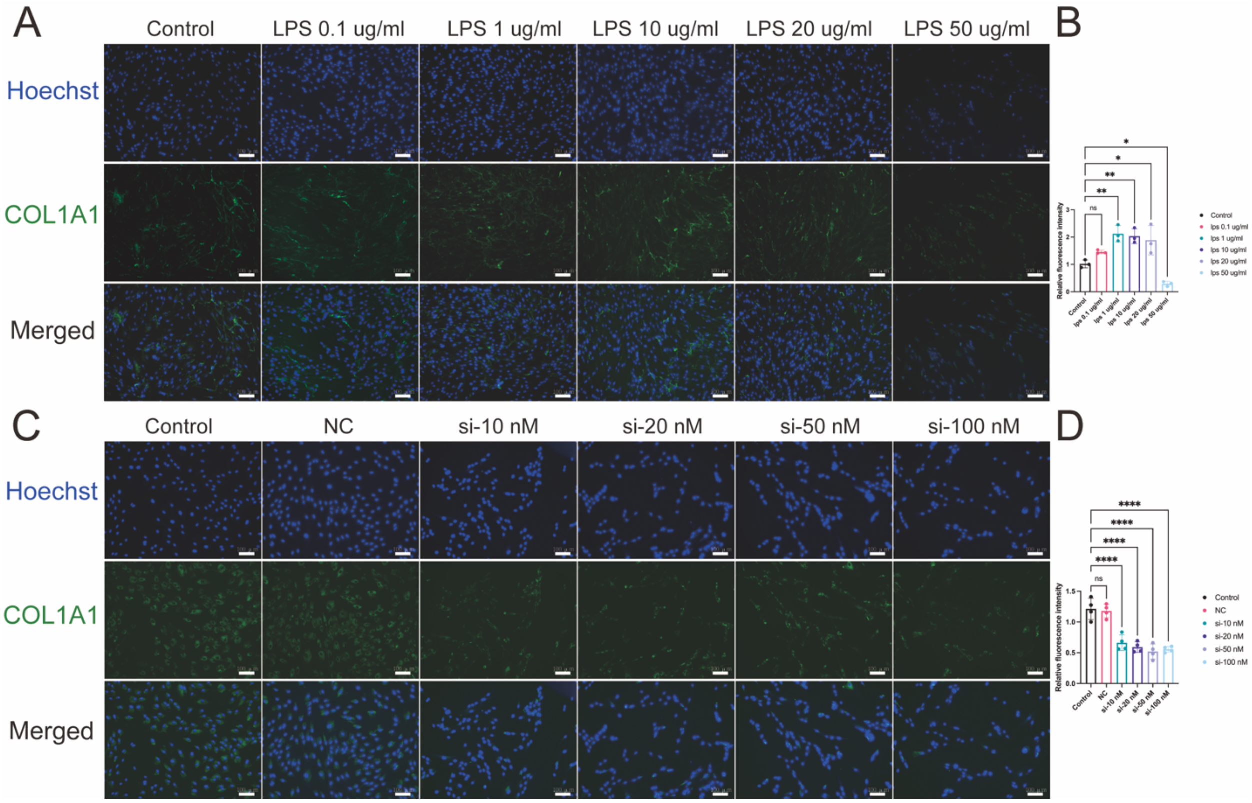

Figure S9. Effect of LPS or knockdown of ITIH4 on collagen I of fibroblasts. (A-B) Fibroblasts were subjected to 0.1-50 μg/ml LPS treatment for 48 h. Representative images and quantitative analysis of synthesis of COL1A1 via immunofluorescence analysis. (n=3). **(C-D)** Fibroblasts were transfected with 10-100 nM ITIH4 siRNA for 48 h. Representative images and quantitative analysis of synthesis of COL1A1 via immunofluorescence analysis. (n=3). Scale bar = 100 μm. ns, not significant; * $P < 0.05$; ** $P < 0.01$; *** $P < 0.001$; **** $P < 0.0001$
